# Primary angiitis of the CNS and ANCA-associated vasculitis: from pathology to treatment

**DOI:** 10.1007/s00296-023-05461-9

**Published:** 2023-09-30

**Authors:** Alaa Sherri, Mohamad Mahdi Mortada, Joanna Makowska, Anna Lewandowska-Polak

**Affiliations:** 1 Department of Rheumatology, Medical University of Lodz, Łódź, Poland; 2 Department of Immunology and Allergy, Medical University of Lodz, Łódź, Poland

**Keywords:** Primary angiitis of the central nervous system (PACNS), ANCA-associated vasculitis (AAV) with central nervous system involvement, Vasculitis of the central nervous system (CNS), Diagnostic workup and treatment

## Abstract

Vasculitis of the central nervous system can be a localized process, such as primary angiitis of the central nervous system (PACNS), or systemic vasculitis, such as ANCA-associated vasculitis (AAV). Since both conditions share neurological manifestations, the following review will discuss the neurological aspects of both. This review aims to provide a comprehensive comparison of the pathogenesis, clinical manifestation and assessment, diagnostic workup, and treatment protocol for both PACNS and AAV with central nervous system involvement. To provide a comprehensive comparison and update, a literature review was conducted using PubMed and Ovid databases (Embase and Medline). Then, the references were retrieved, screened, and selected according to the inclusion and exclusion criteria. PACNS and AAV share similarities in clinical presentation and neurological symptoms, especially in terms of headache, focal deficits, and cognitive impairment. Additionally, both conditions may exhibit similarities in laboratory and radiological findings, making brain biopsy the gold standard for differentiation between the two conditions. Moreover, the treatment protocols for PACNS and AAV are nearly identical. Comparing PACNS and AAV with CNS involvement highlights the similarities in clinical presentation, radiological findings, and treatment protocols between the two conditions. Further research should focus on establishing a practical diagnostic protocol.

## Introduction

Vasculitides are a cluster of different disorders affecting multiple systems in the body. They are characterized by the hallmark of inflammation of the blood vessels that leads to reactive damage to vessel structure, loss of vessel integrity, vessel stenosis, ischemia, and necrosis [1, 2] The revised International Chapel Hill Consensus Conference on the nomenclature of systemic vasculitides (CHCC 2012) introduced the terms “primary” and “secondary” vasculitis and classified the former as large, medium, small, and variable vessel vasculitis. Additionally, it emphasizes the localization, classifying vasculitis as either “single-organ vasculitis” or “vasculitis associated with systemic disease” [3, 4]. In accordance, central nervous system vasculitis is classified into localized primary central nervous system vasculitis, also called primary angiitis of the central nervous system (PACNS), and systemic secondary central nervous system vasculitis, such as AAV of the central nervous system [2, 5, 6]. According to Salvarani et al. [7], primary angiitis predominantly affects small and medium vessels, such as leptomeningeal and parenchymal arterial vessels, and causes medium vessel vasculitis (MVV) and small vessel vasculitis (SVV) in approximately 45–69% and 23–30% of cases, respectively. The involvement of small and medium vessels is a common feature of PACNS and AAV, which justifies our review’s choice to compare these two subtypes of central system vasculitis. Vasculitis of the central nervous system can manifest with diverse clinical presentations, and according to Nehme et al. [8], it is sometimes impossible to promptly diagnose primary angiitis without further investigations, such as CNS biopsies, radiological findings, and comprehensive clinical assessment. The diagnosis and treatment of both primary and secondary vasculitis pose an enormous challenge to physicians due to the absence of a classical presentation and the presence of disease mimickers for both conditions [6, 8]. Furthermore, the similarities in neurological manifestations between the two conditions and the lack of standardized radiological findings specific to vasculitis led to the absence of an ideal diagnostic workup for early detection [4, 9, 10].

This review aims to provide a comprehensive overview and comparison of the pathogenesis, clinical manifestations, and assessment of PACNS and AAV with CNS involvement. The review also aims to highlight a proposed workup for the differentiation and diagnosis of both subtypes based on clinical and radiological findings.

## Search strategy and methods

**Fig. 1:**
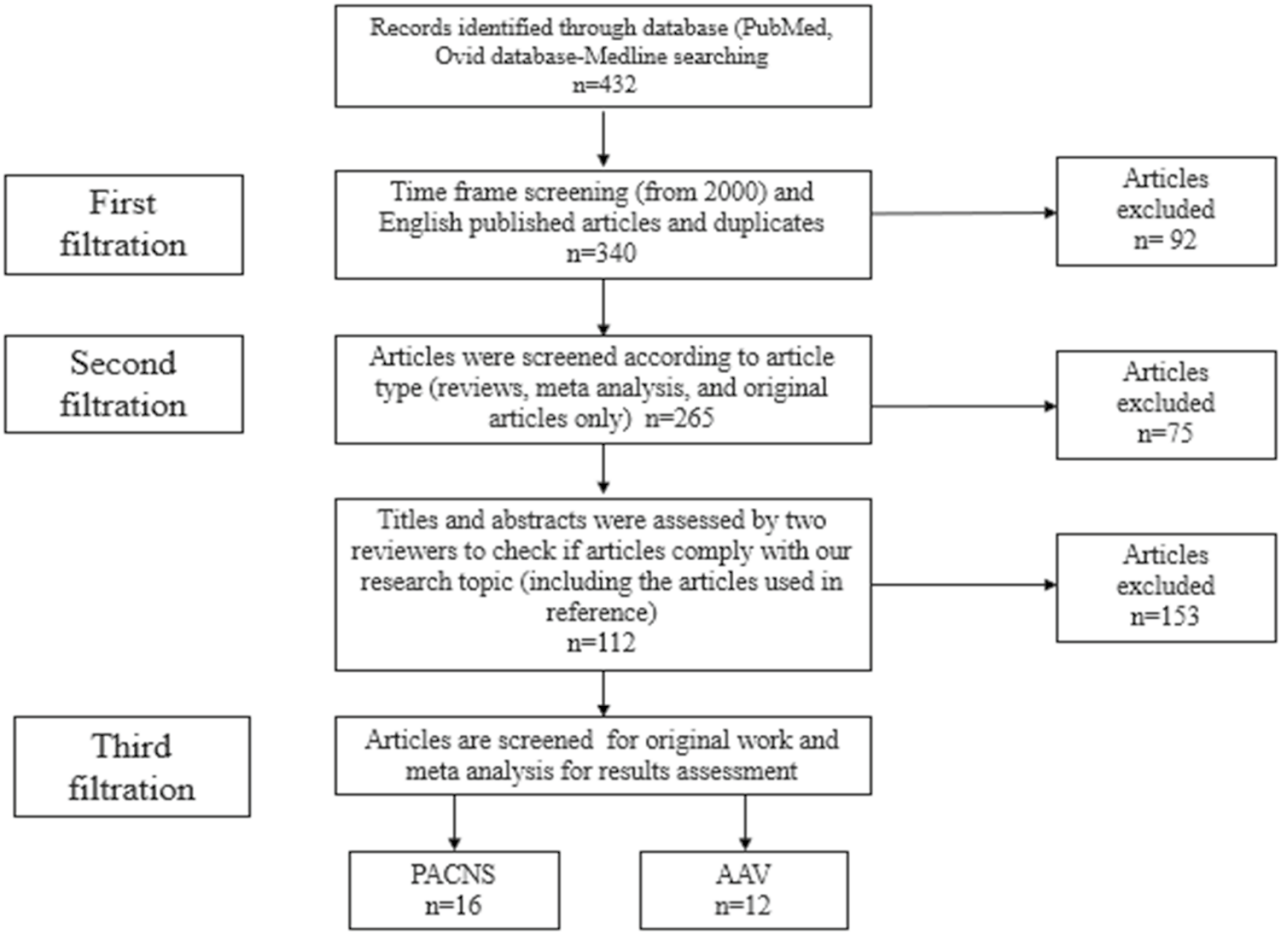
Flowchart showing the article selection criteria

To provide a comprehensive comparison and update, a literature review was conducted using PubMed and Ovid databases (Embase and Medline) for keywords such as ANCA-associated vasculitis with CNS involvement, primary angiitis of central nervous systems, vasculitis of the central nervous system, diagnostic workup, and treatment (Table 1). A total of 432 publications, including those listed in the references, dated from 1989 to 2022 were retrieved and screened. Then, after excluding non-English publications, outdated studies (before 2000), duplicates, publications that did not comply with the aim of our review and nonoriginal articles. 12 and 16 original research articles for ANCA vasculitis and PACNS, respectively, were collected. A flowchart that shows article selection criteria is presented in Fig. 1.

**Table 1:** PubMed/Medline PICO search strategy using MeSH keywords

PICOS	Description	MeSH Keywords
Population (P)	Patients with central nervous system Vasculitis (primary angiitis of the central nervous system or ANCA-associated Vasculitis)	#1 (“primary angiitis of the central nervous system” [MeSH] OR “ANCA associated vasculitis with central nervous system involvement” [MeSH] or “vasculitis of the central nervous system” [MeSH])
Intervention (I)	Diagnostic workup and treatment protocol for primary angiitis of central nervous system or ANCA-associated vasculitis	#2 (“diagnostic workup” [MeSH] and (“primary angiitis of central Nervous system or ANCA associated vasculitis with central nervous system involvement”) or (“treatment” [MeSH] and [(“primary angiitis of central Nervous system” or “ANCA associated vasculitis with central nervous system involvement”)]
Compare (C)	Comparing pathogenesis, clinical manifestation, assessment, and diagnostic workup between PACNS and AAV with CNS involvement	
Outcome (O)	Establishing similarities in clinical presentation between PACNS and AAV and understanding the gold standard for differentiating between the two conditions. Additionally, introducing a suggested diagnosis protocol	
Search strategy	#1 AND #2

## Comparison of pathology, classification, and characteristics

CNS vasculitis is classified into primary and secondary vasculitis, in which both sub classifications hold many vasculitis disorders. PACNS is a localized, rare, and uncommon form of vasculitis affecting the central nervous system, as it is restricted only to the brain and the spinal cord [11]. The etiology of PACNS is not well understood, and many scientists label it idiopathic; however, current research suggests that infectious triggers could be the cause of this type of inflammation, such as varicella zoster virus (VZV) and mycoplasma-like organisms [7]. Studies show that the infiltration of T lymphocytes around cerebral vessels reinforces the role of memory T cells in the pathogenesis of vasculitis [12]. However, the hallmark of this disease is represented by transmural inflammation of the cerebral vessels along with features of parenchymal necrosis and infarction in later stages [13]. In most cases, the clinical presentation of PACNS was postulated to be either due to both immune complex deposition and cell-mediated immunity inflammation, or to necrosis of vessel walls [6]. PACNS can be classified by using two aspects: the size of the vessel and histopathology. Since pathological findings affect both medium and small vessels, PACNS is divided into primary angiitis affecting large, medium, and small vessels [13]. The other classification is based on the histopathological patterns found in biopsies of different PACNS subsets, and they are granulomatous, lymphocytic, and necrotizing histological patterns of vasculitis [7]. 

AAV, labeled both CNS-relatable primary systemic vasculitis and secondary CNS vasculitis, is believed to predominantly affect small vessels such as arteries, arterioles, veins, and venules [14]. These necrotizing diseases share important common features, such as inflammation of the airways, progressive and pauci-immune glomerulonephritis, and increased levels of anti-neutrophil cytoplasmic antibody (ANCA), which is a key identifier in this type of vasculitis [15]. AAVs are sub classified into granulomatosis with polyangiitis (GPA), microscopic polyangiitis (MPA), and eosinophilic granulomatosis with polyangiitis (EGPA). It is important to note that there are two types of antigens detected in AAV: proteinase 3 (anti-PR3) and myeloperoxidase (anti-MPO) [3]. Accordingly, anti-proteinase 3 antibodies are more often detected in GPA, while anti-myeloperoxidase (MPO) is associated with MPA. As a distinct type, EGPA can be associated with both types of antibodies [15]

It is believed that the nervous system is affected in AAV, and the prevalence is estimated to be between 22–54% and 45–72% among patients with GPA and MPA, respectively [16–18]. The mechanism of AAV manifestation in the central nervous system is postulated to be either granulomatous inflammation originating in the sinonasal structure, which can invade the surrounding structures, CNS localized granulomatous infection, or vasculitis of the brain and spinal cord [14]. However, many studies show that CNS manifestations are relatively rare and are present only among 15% of AAV patients [19]. Additionally, the prevalence of CNS and peripheral nervous system PNS manifestations varies when compared between GPA, MPA, and EGPA. Research estimates that the highest prevalence of PNS involvement is described among the EGPA group (up to 70%) and to a lesser extent in GPA and MPA patients (approximately 20–50% for both groups) [20, 21]. The prevalence, subtypes, histopathology, and pathophysiology of CNS vasculitis are summarized in Table 2.

**Table 2:** A summary of the prevalence, subtypes, histopathology, and pathophysiology of CNS vasculitis [1, 5, 22–24]

CNS vasculitis	Prevalence	Forms/subtypes	Histopathology	Pathophysiology
Primary angiitis of the central nervous system (PACNS)	2.4/1000000 M = W	Angiogram negative biopsy positive primary CNS vasculitis	Granulomatous presentation: mononuclear inflammation and granulomas with multinucleated cells	The etiology is unknown Infectious agents like varicella zoster virus (VZV) are believed to trigger the inflammation panel Studies show the infiltration of T lymphocytes around cerebral vessels which stresses the role of memory T cells in the pathogenesis of vasculitis and suggests that primary CNS vasculitis is a result of an antigen-specific immune response
		Rapidly progressive primary CNS vasculitis	Granulomatous or necrotizing, the latter which presents transmural fibrinoid necrosis	
		Primary central nervous system Vasculitis with intracranial hemorrhage	Necrotizing vasculitis presentation	
		Primary CNS vasculitis presents with a solitary tumor-like mass lesion	Deposition of amyloid β	
		Angiography-negative primary CNS vasculitis In children	Lymphocytic inflammation with the presence of plasma cells and features of vessel destruction	
		Leptomeningeal enhancement in Magnetic Resonance imaging (MRI)	Granulomatous pattern presentation	
		Amyloid-β-related cerebral angiitis (ABRA)	Deposition of amyloid β with features of the granulomatous presentation	
ANCA-associated vasculitis (AAV)	2.1–14.4/1000000	Granulomatosis with Polyangiitis (GPA)	Necrotizing or granulomatous pattern vasculitis. presence of lympho-monocyte inflammatory infiltrate, with multinucleated giant cells and fibroblastic proliferation	Multifactorial etiology and pathogenesis (environmental exposure, genetic predisposition, infection, injury, loss of B-cell and T- cell tolerance, and production of ANCA) Characterized the absence or a paucity of immunoglobulin deposition in vessel walls
	2.4–10.1/1000000	Microscopic Polyangiitis (MPA)		
	0.5–6.8/1000000	Eosinophilic Granulomatosis with Polyangiitis (EGPA)	Eosinophil-rich infiltrates with necrotizing and/or granulomatous pattern vasculitis	

An increase in incidence was reported for both diseases in several research studies [25]. For instance, a case study reported the presentation of AAV as an isolated intraparenchymal mass with a clinical manifestation limited only to the CNS [25, 26].

## Comparison of CNS clinical manifestations

### Clinical findings in AAV

The neurological findings can vary between the central and peripheral nervous systems. It is important to understand the scope of clinical and radiological involvement of AAV, since many clinical and radiological manifestations depend on the affected anatomical structure. The scope of the disease can affect the brain parenchyma, meninges, pituitary gland stalk, and spinal cord, in addition to non-CNS entities such as cranial nerves, orbits, and Sino-nasal structures [27]. Early signs of AAV of the CNS include ischemic infarction and intracranial hemorrhage that can either be restricted to the brain parenchyma or extend to the subarachnoid space and are often resistant to antiplatelet therapy [28]. Some papers reported relapsing cases of subarachnoid hemorrhage, and in a different case reported by Esfahani et al., intracranial hemorrhage appeared in a patient after administering tissue plasminogen activator (tPA) for acute ischemic stroke, which stresses the need of cautious diagnosis to exclude AAV [29, 30]. Cognitive impairment is another manifestation of brain parenchyma involvement and is characterized by a cognitive decline in abstract reasoning, memory, and attention [31]. Later outcomes of AAV of the CNS is posterior reversible encephalopathy syndrome (PRES) in the brain parenchyma, and it appears with symptoms like encephalopathy, headaches, and visual disturbances [27]. Hypertrophic pachymeningitis (HP), which can involve both the brain meninges and spinal cord, is also described among AAV patients. Studies have shown that AAV patients with HP present higher frequencies of headaches and cranial nerve palsy than those without HP [27, 32]. Moreover, the study by Shimojima et al. demonstrated that the prevalence of HP was lower in the early stages of AAV, which suggests that HP involvement is more common in the late stages of AAV and that CNS manifestation might prevail in the later stages of AAV [32]. Pituitary gland involvement is also a common presentation of AAV of the CNS that presents as either hypopituitarism, diabetes insipidus, or visual defects due to compression of the optic chiasm [33, 34].

### Clinical presentation variation based on subtypes and ANCA serology

Manuscripts on AAV manifestation in the central nervous system as well as the clinical assessment of AAV patients are presented in Table 3.

**Table 3:** Original research articles on ANCA-associated vasculitis manifestation in the central nervous system [14, 19, 35–37]

Publications	De Luna et al.	Wang et al.	Fragoulis et al.	Lubas et al.	Ma et al.
Type	Case‒control	Cohort	Cohort	Cohort	Cohort
Form of the studied AAV	GPA	EGPA	GPA	GPA	GPA + MPA
Population	35	19	9	37	29
Headaches	23/35 (66%)	5/19 (71.4%)	3/9 (33.3%)	8/37 (21.6)	5/18
Motor impairment	11/35 (31%)	2/19 (10.5%)	3/9 (33.3%)	10/37 (27%)	3/18
Seizures	NA	1/19 (7.7%)	2/9 (22.2%)	0%	3/18
Sensory impairment	15/35 (43%)	1/19 (7.7%)	1/9 (11.1%)	10/37 (27%)	5/18
Gait	NA	NA	1/9 (11.1%)	9/37 (24.3%)	NA
Diabetes insipidus	2/35 (6%)	NA	NA	NA	1/18
Psychiatric/mood disorders	3/35 (9%)	NA	NA	3/14 (21.4%)	NA

Headaches are thought to be the first and predominant manifestation of CNS vasculitis, especially in GPA patients, and this nonspecific presentation can delay the diagnosis [18]. Among the assessed research articles, a case study by Caramaschi et al. reported that CNS symptoms such as headaches, hearing loss, and olfactory deficiency were predominant [38]. Moreover, the most frequent clinical presentations among all forms of AAV patients were sensorimotor impairment and headaches, while other presentations of symptoms such as seizures, gait, and hearing loss were variable among research on different subsets [14, 35–37]. Previous results can be explained by the different forms of AAV involved, which have a variation in the appearance of clinical symptoms. GPA mainly affects three important structures of the CNS: the meninges, pituitary gland, and cerebral vasculature, while EGPA and MPA lead mainly to cerebral infarctions and intracerebral hemorrhage [18, 25, 39]. The pituitary gland is estimated to be affected in 1.1–1.3% of GPA patients [18]. Hypophysis of the pituitary gland in those patients can cause abnormalities and dysfunction of both the posterior and anterior pituitary glands. Diabetes insipidus patients were reported in the finding of both Ma et al. and De Luna et al. The manifestation of diabetes insipidus is anatomically postulated to contribute to the pathogenesis and mechanism of AAV, especially in GPA, in which its granulomatous inflammation can invade Sino-nasal surrounding structures [39]. In addition to diabetes insipidus, inflammation can lead to secondary gonadism, secondary hypothyroidism, secondary adrenal insufficiency, and secondary growth hormone deficiency [40]. GPA subtype of AAV can also cause compression of the pituitary stalk, which can lead to hyperprolactinemia, galactorrhea, and visual disturbances [41]. Psychiatric disorders were also present among the assessed patients and manifested as depression, delirium, and cognitive impairment [28, 39]. The previous impairments are most likely linked to cerebral ischemic and hemorrhagic vasculitis damage, in addition to other presentations, such as transient ischemic attack (TIA), seizures, and cortical blindness [18]. The involvement of GPA in the meninges, especially granulomatous inflammatory thickening of the dura, has been proven to be linked to symptoms such as cerebellar ataxia, seizures, and cranial neuropathies. Chronic hypertrophic pachymeningitis is linked to ANCA serology and presented as an outcome for both GPA and MPA similarly [18]. Spinal cord involvement is more common among MPA patients than the other subtypes [27].

### Clinical findings in PACNS

The clinical symptoms of PACNS are variable, and to help in recognizing the disease, three suggested clinical presentations were described [42]: (1) acute or subacute encephalopathy that can progress from confusion to drowsiness and coma; (2) multiple sclerosis presentation or mimicking presentation with atypical features, remitting and relapsing course such as optic neuropathy and brainstem episodes. In addition to symptoms such as seizures, persistent headaches, stroke-like episodes, and encephalopathy; and (3) intracranial mass lesions with headaches and focal signs [10, 43–63]. To interpret these presentations, it is suggested that if large and medium vessels are involved, stroke is most likely the manifestation. If small vessels are involved, encephalopathy, cognitive dysfunction, and seizures are the main manifestations [13]. In a systematic review written by Sarti et al., 24 case series were grouped, and the clinical manifestation frequency was assessed. This case series had 585 patients diagnosed with PACNS, with 41% of the cases confirmed with a biopsy [11]. According to Sarti et al., headaches were present in 57.1% (320/560) of patients, making it the first and most common symptom among all PACNS cases [11]. However, thunderclap headaches should be excluded from this criterion, as it was shown that it is a manifestation of reversible cerebral vasoconstriction syndrome [13]. Cognitive impairment was reported in 42.7% (190/445) of patients, while stroke and TIA were present in 44.4% (340/564) of patients. Sundaram argues that the acute onset of focal neurological deficits is due to strokes and TIA [13]. Other presentations, such as seizures, impaired level of consciousness, and mood disorders, accounted for 26.9%, 30.6%, and 20.9%, respectively. In other reports, stroke-like symptoms, seizures, frontal lobe dementia, and ataxia were the first reported manifestations of PACNS rather than headaches [64–66].

## Comparison of radiological findings

### Radiological findings in AAV

For patients suspected to have central nervous system vasculitis, it was recommended by the American College of Radiology Appropriateness Criteria (ACR) to use head magnetic resonance angiography (MRA) with or without contrast [67]. A summary of the most common MRI findings among AAV patients is as follows [19, 27, 35]:

- Cerebral ischemia lesion presenting as ischemic infarction and extensive white matter lesions or hyper intense in T2FLAIR images.
- Hemorrhagic lesions such as subarachnoid, basal ganglia, and subdural hemorrhage
- Enlargement of hypophysis with infundibular thickening or patchy pituitary masses
- Granulomatosis of the brain and spinal cord
- Pachymeningitis is characterized by thickened dura matter.
- Ophthalmic/orbital masses
- Paranasal thickening of the sinus wall or “ground-glass” material in the lumen
- Changes in the spinal cord or spinal cord hemorrhage

Research has shown that cerebral ischemia is a predominant finding among AAV patients, as similarly confirmed in research conducted by Ma et al.; 24/29 patients showed cerebral ischemia on MRI [19]. Features of vascular alternating narrowing and dilation (VAND) were detected among GPA patients in the study performed by Lubas et al., suggesting that VAND can occur as a feature of vasogenic alteration in AAV [37]. In later stages of AAV of the CNS, vasogenic edema involving the bilateral parietal–occipital region might be found during screening and is consistent with PRES [27].

### Radiological findings of PACNS

Radiological findings of PACNS are diverse and can be measured by several techniques depending on the size of vessels and the symptom manifestation. However, identifying features that are consistent with PACNS is challenging since many are common among the different subtypes of vasculitis. For example, in a study conducted by Arnet et al., luminal stenosis was a common finding among all types of vasculitis [68]. It is important to differentiate between radiological features that are consistent with other diseases while building a differential diagnosis. For example, changes and occlusions in the fusiform vessel wall are considered shared characteristics between PACNS and polyarteritis nodosa, giant cell arteritis, Sjogren syndrome, and infectious vasculitis [68].

## Comparison of current diagnosis and treatment strategies

### Diagnosis of AAV patients

The diagnosis of AAV of the CNS is composed of 3 clinical steps, laboratory, and radiological assessment. Starting with laboratory assessment, a very detailed workup should be ordered for patients who are suspected to have AAV. ANCA serology is a very important parameter to be assessed, although it is not a confirmatory or diagnostic parameter for AAV of the CNS. This is because a seronegative ANCA immunoassay is also a finding among many GPA and MPA patients and because ANCA can be positive in other vasculitis diseases, systemic disorders, infections, drugs, gastrointestinal diseases, and malignancies [27, 69]. A high-quality antigen immune assay for both anti-MPO and anti-PR3 is highly recommended, especially because it allows physicians to have more insight into the presentation of the disease and characteristics related to remission and relapses. Patients diagnosed with MPA have a lower relapse rate than patients diagnosed with GPA [14]. Inflammatory markers are very important for testing AAV, and its findings present as elevation of both erythrocyte sedimentation rates (ESR) and C-reactive protein (CRP), leukocytosis, and thrombocytosis [35, 70, 71]. Physicians suspecting AAV are also recommended to assess kidney function and autoimmune panels to assess mimics for differential diagnosis [27]. Since many cases of AAV present pituitary involvement, it is recommended to carry out an endocrine panel related to pituitary hormones, especially if an abnormal pituitary screening is obtained [33, 34]. Analysis of cerebrospinal fluid (CSF) is an important step in the diagnosis of AAV of the CNS, and physicians expect to find parameters of meningitis, such as protein > 0.40 g/l, WBC > 10/mm^3^, and glucose < 2.8 mmol/L [35]. Histopathology or biopsy of the CNS is the gold standard in the diagnosis of AAV, and several research works had limitations in their findings for not including CNS biopsy as a diagnostic tool [14]. Pathologists aim to find a necrotizing or granulomatous pattern, lympho-monocyte inflammatory infiltrate, multinucleated giant cells, and fibroblastic proliferation [5, 7, 72]. It is important to highlight that a negative biopsy result does not exclude the probability of AAV due to the segmental quality of the lesions present in the CNS [27]. MRI of the brain and spinal cord needs to be assessed for any abnormalities that were discussed in the previous section. Some scores are used to assess the degree of the disease manifestation, such as the Birmingham vasculitis activity score (BVAS), a clinical index of disease activity based on clinical evaluation of nine organ systems [73].

### Diagnosis of PACNS

The investigation for PACNS is controversial, and until today, there are no clinical, laboratory, or radiological findings that are 100% confined to this condition. This imposes a challenge on physicians to make the correct diagnosis and exclude all the mimics and variants of the disease. The first diagnostic approach was proposed by Calabrese and Mallek in 1988 and included the following criteria [5, 74]:

1. Patients presenting with a history or clinical findings of an acquired neurological deficit that cannot be linked to a known origin on assessment.
2. Having a cerebral angiography or a cerebral biopsy showing classic features of vasculitis
3. No other evidence of systemic vasculitis or any other disorder to which the radiological and pathological features can be consistent.

In 2009, Birnbaum et al. proposed a modification to these criteria by highlighting the most likely mimics of PACNS [9]. They also proposed the level of certainty that is divided into definite, which is when tissue biopsy specimen results are consistent with the diagnosis of PACNS, and probable, if the result findings are highly probable of PACNS on angiogram, MRI, and CSF but without a confirmatory tissue biopsy [5, 9]. Therefore, after symptoms are suspected, the first step is to obtain an MRI, which in most cases of PACNS is pathological. Then, cerebral angiography or biopsy is indicated with other laboratory investigations to rule out secondary causes of CNS vasculitis and mimics of PACNS.

Laboratory parameters are needed, especially the laboratory workup focusing on inflammation and antibody-mediated disease. Assessment of CSF is also important in diagnosing PACNS and in excluding other possibilities of vasculitis, such as infections and neoplasms [13]. Pathological CSF findings that are consistent with PACNS were described as elevated cell count (pleocytosis) with or without an increase in protein levels, and the following was present in 43.5% of patients diagnosed with PACNS [75, 76]. Oligoclonal banding may also be found in the sample [77]. Neuroimaging is variable, and each technique has a different indication. CT scans are usually indicated for the detection of single or multiple infarcts and intracerebral hemorrhages. On the other hand, Magnetic resonance imaging (MRI) is indicated for the observation of parenchymal and meningeal lesions. Magnetic resonance angiography (MRA) helps detect large and medium-sized vessel involvement, while cerebral angiography (CA) or conventional 4-vessel digit subtraction angiography is considered a confirmatory imaging technique for PACNS. Biopsy is still the gold standard in confirming PACNS.

### Suggested diagnostic criteria for PACNS

A suggested diagnostic criterion was proposed by Sarti et al. [11]. First, they calculated the median frequencies for both clinical and radiological features present in the case studies that they worked on. Accordingly, the median frequency for clinical features was 42.7% and that for radiological features was 46.6%. Second, they defined the major and minor clinical and radiological features based on whether they were greater than or less than the calculated frequencies. The major clinical features consisted of headaches, stroke, cognitive impairment, and focal neurological deficits, while the minor criteria consisted of seizures, altered levels of consciousness, and psychiatric disorders. For the neuroradiological findings, the major criteria consisted of multiple parenchymal lesions, parenchymal or meningeal contrast, enhancement vessel abnormalities, and vessel wall contrast enhancement. The minor features, on the other hand, consisted of parenchymal or subarachnoid hemorrhages and single parenchymal lesions.

Third, they developed 7 possible combinations of these features, but only the first two A and B combinations were consistent with the Calabrese and Mallek criteria and verified in all 32 case studies and, thus, can be taken into consideration in the future evaluation of patients. The first combination A consists of one clinical (major + minor) feature and one major neurological feature. On the other hand, combination B consists of two clinical features (at least one major feature with one of the major neurological features) [11]. However, this study has many limitations, and more research is needed to validate the implementation of such proposed criteria.

## Management

### Management of AAV patients

The management of AAV patients is based on the remission-induction phase, followed by a maintenance phase [78]. To differentiate between different research outcomes, Ma et al. defined 3 aspects of management findings with the Birmingham vasculitis activity score (BVAS) new/worse. This score defined complete remission as the absence of disease activity that needs stable maintenance immunosuppressive therapy, while partial remission was defined as a minimum reduction in the disease activity score by half without the appearance of new manifestations [14]. Relapse, however, was defined as the reoccurrence of new disease activity symptoms indicating inflammation, with the patient having achieved remission before [14]. Usually, the management regimen in remission induction consists of a combination of glucocorticoids with cyclophosphamide. Generally, the remission-induction phase is started using a highly potent immunosuppressant (e.g., cyclophosphamide) with glucocorticoids for a duration of up to three or four months, followed by the second phase, which is maintenance using an immunosuppressant (e.g., azathioprine) with a low-dose glucocorticoid for a duration of at least 1.5 to 2 years [79]. In a study performed by Suka et al., the use of cyclophosphamide and prednisone was assessed, and during the 18 months of follow-up, there was a notable improvement in health-related quality of life and significant improvement in BVAS score [80]. It is worth mentioning that among AAV patients in whom there is no threat of organ damage, methotrexate, which is an immunosuppressant with medium potency, can be used for the remission-induction phase [81]. New studies have introduced methotrexate as a good candidate for the maintenance phase [78].

Additionally, one newly emerged treatment option to be used in both the remission-induction and maintenance phases is rituximab (RTX) [21, 28, 82]. In the research work of Stone et al., 197 AAV patients with either GPA or MPA were included to compare the efficacy of the rituximab and cyclophosphamide regimens in the patient’s treatment [82]. The results of the study indicated that the rituximab regimen showed higher efficacy than the cyclophosphamide regimen with the same prevalence regarding the occurrence of adverse effects. For pregnant women, it is recommended to plan accordingly. Both male and female patients are prohibited from taking any teratogenic drugs, such as cyclophosphamide or methotrexate, 3–4 months before conception; however, current research has found that cyclophosphamide treatment during the 2nd and 3rd trimesters is relatively safe [83]. For spinal cord microscopic polyangiitis, Yang et al. reported a case where the resection of the intramedullary lesions proved to be effective in decreasing the neurological manifestation. The previous outcomes give hope in integrating surgical treatment into the management protocol of AAV with CNS involvement [84].

### Management of PACNS patients

Similar to AAV patients, the therapeutic procedure of PACNS patients is based on two parts, remission induction and maintenance, with specific drugs used in each of the two phases. In one of the studies, 167 PACNS patients were included, and therapeutic options were compared. Although induction therapy consisting of the use of cyclophosphamide and glucocorticoids showed equal efficacy to therapy with glucocorticoids alone, the combined therapy was associated with a lower occurrence of relapses [43]. In another study, 52 patients with PACNS were included, and the results indicated that patients managed with combination therapy tend to show better treatment outcomes [85]. Usually, after four–six months into the remission-induction phase, the maintenance phase is conducted using several possible agents: azathioprine, mycophenolate mofetil, and methotrexate [7, 86]. Several new research studies have shown that biological agents (e.g., rituximab) can also be used in the remission-induction phase [87, 88]. In Table 4, agents used to treat PACNS patients with their dosage and route of administration will be demonstrated.

**Table 4:** Summary of agents used to treat PACNS patients [7, 85–89]

	Cyclophosphamide	Oral (2 mg/kg/day) Monthly IV pulse (starting at 750 mg/m^2^)
Remission induction	Corticosteroids 1. Prednisone Or 2. Methylprednisolone	1.Oral (1 mg/kg/day) Or 2. IV pulse (1000 mg daily for 3–5 days)
	Rituximab	375 mg/m2/week for 4 weeks Or 2 IV doses of 1 g each, administered 2 weeks apart
Maintenance	Azathioprine	1–2 mg/kg/day
	Methotrexate	2–25 mg/week
	Mycophenolate mofetil	1–2 g/daily

## Conclusion

AAV with CNS involvement has been shown to have similar clinical presentation, radiological findings, and treatment protocols to PACNS. Therefore, primary and secondary central nervous systems are broad classifications that need more definite protocols for diagnosis and treatment due to the presence of different subtypes and serological variants. Reasonable diagnostic protocols need to be evaluated in future research.

## References

[1] Mansueto G, Lanza G, Fisicaro F (2022). Central and peripheral nervous system complications of vasculitis syndromes from pathology to bedside: part 1—central nervous system. Curr Neurol Neurosci Rep.

[2] Mumtaz S, Fowler MR, Gonzales-Toledo E, Kelley RE (2005). Central nervous system vasculitis pathogenesis, immunology, and clinical management.

[3] Jennette JC, Falk RJ, Bacon PA, et al (2013) 2012 Revised International Chapel Hill consensus conference nomenclature of vasculitides. In: Arthritis and Rheumatism. pp 1–11 10.1002/art.37715 23045170

[4] Jennette JC (2013). Overview of the 2012 revised international chapel hill consensus conference nomenclature of vasculitides. Clin Exp Nephrol.

[5] Beuker C, Schmidt A, Strunk D (2018). Primary angiitis of the central nervous system: diagnosis and treatment. Ther Adv Neurol Disord.

[6] Sánchez-Román E, Monternach-Aguilar F, Reyes-Vaca JG, Rodríguez Leyva I (2021). Challenging presentation of primary vasculitis of the central nervous system. Cereb Circ Cogn Behav.

[7] Salvarani C, Brown RD, Hunder GG (2012). Adult primary central nervous system vasculitis. Lancet.

[8] Nehme A, Boulanger M, Aouba A (2022). Diagnostic and therapeutic approach to adult central nervous system vasculitis. Rev Neurol (Paris).

[9] Birnbaum J, Hellmann DB (2009). Primary angiitis of the central nervous system. Arch Neurol.

[10] Vera-Lastra O, Sepúlveda-Delgado J, del Cruz-Domínguez MP (2015). Primary and secondary central nervous system vasculitis: clinical manifestations, laboratory findings, neuroimaging, and treatment analysis. Clin Rheumatol.

[11] Sarti C, Picchioni A, Telese R (2020). “When should primary angiitis of the central nervous system (PACNS) be suspected?”: literature review and proposal of a preliminary screening algorithm. Neurol Sci.

[12] Iwase T, Ojika K, Mitake S (2001). Involvement of CD45RO+ T lymphocyte infiltration in a patient with primary angiitis of the central nervous system restricted to small vessels. Eur Neurol.

[13] Sundaram S, Sylaja P (2022). Primary angiitis of the central nervous system—diagnosis and management. Ann Indian Acad Neurol.

[14] Ma TT, Li ZY, Geng YS (2020). Central nervous system involvement in patients with antineutrophil cytoplasmic antibody–associated vasculitis: a study of 29 cases in a single Chinese center. Clin Rheumatol.

[15] Nocton JJ (2017). Usual and unusual manifestations of systemic and central nervous system vasculitis. Pediatr Clin North Am.

[16] Sada K, Yamamura M, Harigai M (2014). Classification and characteristics of Japanese patients with antineutrophil cytoplasmic antibody-associated vasculitis in a nationwide, prospective, inception cohort study. Arthritis Res Ther.

[17] Agard C, Mouthon L, Mahr A, Guillevin L (2003). Microscopic polyangiitis and polyarteritis nodosa: how and when do they start?. Arthritis Rheum.

[18] Graf J (2017). Central nervous system disease in antineutrophil cytoplasmic antibodies-associated vasculitis. Rheum Dis Clin North Am.

[19] Liu S, Guo L, Fan X (2021). Clinical features of central nervous system involvement in patients with eosinophilic granulomatosis with polyangiitis: a retrospective cohort study in China. Orphanet J Rare Dis.

[20] Bischof A, Jaeger VK, Hadden RDM (2019). Peripheral neuropathy in antineutrophil cytoplasmic antibody-associated vasculitides. Neurol Neuroimmunol Neuroinflamm.

[21] Wludarczyk A, Szczeklik W (2016). Neurological manifestations in ANCA-associated vasculitis - assessment and treatment. Expert Rev Neurother.

[22] Mohammad AJ (2020). An update on the epidemiology of ANCA-associated vasculitis. Rheumatology.

[23] Giannini C, Salvarani C, Hunder G, Brown RD (2012). Primary central nervous system vasculitis: pathology and mechanisms. Acta Neuropathol.

[24] Nilsen AT, Karlsen C, Bakland G (2020). Increasing incidence and prevalence of ANCA-associated vasculitis in Northern Norway. Rheumatology (United Kingdom).

[25] Seror R, Mahr A, Ramanoelina J (2006). Central nervous system involvement in Wegener granulomatosis. Medicine.

[26] Weng J, Du Z, Zhang Y (2022). CNS limited ANCA-associated vasculitis presenting as an isolated intraparenchymal mass. J Neuroimmunol.

[27] Zheng Y, Zhang Y, Cai M (2019). Central nervous system involvement in ANCA-associated vasculitis: what neurologists need to know. Front Neurol.

[28] Treppo E, Binutti M, Agarinis R (2021). Rituximab induction and maintenance in ANCA-associated vasculitis: state of the art and future perspectives. J Clin Med.

[29] Esfahani NZ, Anderson DM, Pieper C, Adams HP (2017). Intracerebral hemorrhage after IV tPA for stroke as early symptom of ANCA-associated vasculitis. eNeurologicalSci.

[30] Xie J, Jia E, Wang S (2022). Relapsing subarachnoid hemorrhage as a clinical manifestation in microscopic polyangiitis: a case report and literature review. Clin Rheumatol.

[31] Mattioli F, Capra R, Rovaris M (2002). Frequency and patterns of subclinical cognitive impairment in patients with ANCA-associated small vessel vasculitides. J Neurol Sci.

[32] Shimojima Y, Kishida D, Ichikawa T (2022). Hypertrophic pachymeningitis in ANCA-associated vasculitis: a cross-sectional and multi-institutional study in Japan (J-CANVAS). Arthritis Res Ther.

[33] Esposito D, Trimpou P, Giugliano D (2017). Pituitary dysfunction in granulomatosis with polyangiitis. Pituitary.

[34] Yong TY, Li JY (2014). Pituitary involvement in granulomatosis with polyangiitis. JCR J Clin Rheumatol.

[35] De Luna G, Terrier B, Kaminsky P (2015). Central nervous system involvement of granulomatosis with polyangiitis: clinical-radiological presentation distinguishes different outcomes. Rheumatology.

[36] Fragoulis GE, Lionaki S, Venetsanopoulou A (2018). Central nervous system involvement in patients with granulomatosis with polyangiitis: a single-center retrospective study. Clin Rheumatol.

[37] Lubas A, Staszewski J, Maliborski A (2022). Vascular and vasogenic manifestations of systemic ANCA-associated vasculitis with renal involvement in non-contrast brain MRI in patients with acute disease onset. J Clin Med.

[38] Caramaschi P, Biasi D, Carletto A, Bambara LM (2003). A case of ANCA-associated vasculitis with predominant involvement of central nervous system. Jt Bone Spine.

[39] Zhang S, Yuan D, Tan G (2019). Neurological involvement in primary systemic vasculitis. Front Neurol.

[40] Peters JE, Gupta V, Saeed IT (2018). Severe localized granulomatosis with polyangiitis (Wegener’s granulomatosis) manifesting with extensive cranial nerve palsies and cranial diabetes insipidus: a case report and literature review. BMC Neurol.

[41] Kapoor E, Cartin-Ceba R, Specks U (2014). pituitary dysfunction in granulomatosis with polyangiitis: the mayo clinic experience. J Clin Endocrinol Metab.

[42] Rice CM, Scolding NJ (2020). The diagnosis of primary central nervous system vasculitis. Pract Neurol.

[43] Salvarani C, Brown RD, Christianson TJH (2015). Adult primary central nervous system vasculitis treatment and course: analysis of one hundred sixty-three patients. Arthritis Rheumatol.

[44] Hajj-Ali RA, Furlan A, Abou-Chebel A, Calabrese LH (2002). Benign angiopathy of the central nervous system: cohort of 16 patients with clinical course and long-term follow-up. Arthritis Rheum.

[45] Salvarani C, Brown RD, Calamia KT (2007). Primary central nervous system vasculitis: analysis of 101 patients. Ann Neurol.

[46] Volcy M, Toro ME, Uribe CS, Toro G (2004). Primary angiitis of the central nervous system: report of five biopsy-confirmed cases from Colombia. J Neurol Sci.

[47] Singhal AB, Topcuoglu MA, Fok JW (2016). Reversible cerebral vasoconstriction syndromes and primary angiitis of the central nervous system: clinical, imaging, and angiographic comparison. Ann Neurol.

[48] MacLaren K, Gillespie J, Shrestha S (2005). Primary angiitis of the central nervous system: emerging variants. QJM Int J Med.

[49] Küker W, Gaertner S, Nägele T (2008). Vessel wall contrast enhancement: a diagnostic sign of cerebral vasculitis. Cerebrovasc Dis.

[50] Molloy ES, Singhal AB, Calabrese LH (2008). Tumor-like mass lesion: an underrecognized presentation of primary angiitis of the central nervous system. Ann Rheum Dis.

[51] Lee Y, Kim J, Kim E (2009). Tumor-mimicking primary angiitis of the central nervous system: initial and follow-up MR features. Neuroradiology.

[52] Kraemer M, Berlit P (2011). Primary central nervous system vasculitis: clinical experiences with 21 new European cases. Rheumatol Int.

[53] Pizzanelli C, Catarsi E, Pelliccia V (2011). Primary angiitis of the central nervous system: report of eight cases from a single Italian center. J Neurol Sci.

[54] Néel A, Auffray-Calvier E, Guillon B (2012). Challenging the diagnosis of primary angiitis of the central nervous system: a single-center retrospective study. J Rheumatol.

[55] Pfefferkorn T, Linn J, Habs M (2013). Black blood MRI in suspected large artery primary angiitis of the central nervous system. J Neuroimaging.

[56] Pourmahmoodian H, GhelichniaOmrani HA, Harrirchian MH (2012). Primary angiitis of the central nervous system. Acta Med Iran.

[57] Coronel-Restrepo N, Bonilla-Abadía F, Cortes OA (2013). Primary angiitis of the central nervous system: a report of three cases from a single Colombian center. Case Rep Neurol Med.

[58] Cosottini M, Canovetti S, Pesaresi I (2013). 3-T magnetic resonance angiography in primary angiitis of the central nervous system. J Comput Assist Tomogr.

[59] Geri G, Saadoun D, Guillevin R (2014). Central nervous system angiitis: a series of 31 patients. Clin Rheumatol.

[60] Oon S, Roberts C, Gorelik A (2013). Primary angiitis of the central nervous system: experience of a Victorian tertiary-referral hospital. Intern Med J.

[61] Niu L, Wang L, Yin X (2017). Role of magnetic resonance imaging in the diagnosis of primary central nervous system angiitis. Exp Ther Med.

[62] Schuster S, Bachmann H, Thom V (2017). Subtypes of primary angiitis of the CNS identified by MRI patterns reflect the size of affected vessels. J Neurol Neurosurg Psychiatry.

[63] Harsha K, Jagtap S, Kapilamoorthy T (2017). CNS small vessel vasculitis: distinct MRI features and histopathological correlation. Neurol India.

[64] Huang Y-J, Zhang L, Mao Y, Nan G-X (2019). Ataxia as the main manifestation of tumor-like primary angiitis of the central nervous system: a case report and literature review. BMC Med Imaging.

[65] Brauns E, Schuind S, Lebrun L (2017). Partial seizures as the first manifestation of primary angiitis of the central nervous system. Eur J Case Rep Intern Med.

[66] Bönstrup M, Ott K, Glatzel M, Magnus T (2016). Frontal lobe dementia syndrome as a first manifestation of primary angiitis of the central nervous system (PACNS). Clin Neurol Neurosurg.

[67] Ledbetter LN, Burns J, Shih RY (2021). ACR appropriateness criteria® cerebrovascular diseases-aneurysm, vascular malformation, and subarachnoid hemorrhage. J Am Coll Radiol.

[68] Arnett N, Pavlou A, Burke MP (2022). Vessel wall MR imaging of central nervous system vasculitis: a systematic review. Neuroradiology.

[69] Weiner M, Segelmark M (2016). The clinical presentation and therapy of diseases related to anti-neutrophil cytoplasmic antibodies (ANCA). Autoimmun Rev.

[70] Kallenberg CGM (2014). Key advances in the clinical approach to ANCA-associated vasculitis. Nat Rev Rheumatol.

[71] Zhang W, Zhou G, Shi Q (2009). Clinical analysis of nervous system involvement in ANCA-associated systemic vasculitides. Clin Exp Rheumatol.

[72] Koike H, Nishi R, Ohyama K (2022). ANCA-associated vasculitic neuropathies: a review. Neurol Ther.

[73] Yumura W, Kobayashi S, Suka M (2014). Assessment of the Birmingham vasculitis activity score in patients with MPO-ANCA-associated vasculitis: subanalysis from a study by the Japanese Study Group for MPO-ANCA-associated vasculitis. Mod Rheumatol.

[74] Calabrese LH, Mallek JA (1988). Primary angiitis of the central nervous system. Medicine.

[75] Becker J, Horn PA, Keyvani K (2017). Primary central nervous system vasculitis and its mimicking diseases—clinical features, outcome, comorbidities and diagnostic results—a case control study. Clin Neurol Neurosurg.

[76] Karaman AK, Korkmazer B, Arslan S (2021). The diagnostic contribution of intracranial vessel wall imaging in the differentiation of primary angiitis of the central nervous system from other intracranial vasculopathies. Neuroradiology.

[77] Berlit P, Kraemer M (2014). Cerebral vasculitis in adults: what are the steps in order to establish the diagnosis? Red flags and pitfalls. Clin Exp Immunol.

[78] Maritati F, Alberici F, Oliva E (2017). Methotrexate versus cyclophosphamide for remission maintenance in ANCA-associated vasculitis: a randomized trial. PLoS ONE.

[79] Holle JU, Gross WL (2013). Treatment of ANCA-associated vasculitides (AAV). Autoimmun Rev.

[80] Suka M, Hayashi T, Kobayashi S (2012). Improvement in health-related quality of life in MPO-ANCA-associated vasculitis patients treated with cyclophosphamide plus prednisolone: an analysis of 18 months of follow-up data from the JMAAV study. Mod Rheumatol.

[81] de Groot K, Schmidt DK, Arlt AC (2001). Standardized neurologic evaluations of 128 patients with Wegener granulomatosis. Arch Neurol.

[82] Stone JH, Talor M, Stebbing J (2000). Test characteristics of immunofluorescence and ELISA tests in 856 consecutive patients with possible ANCA-associated conditions. Arthritis Rheum.

[83] Chaigne B, Guillevin L (2017). Vasculitis for the internist: focus on ANCA-associated vasculitis. Intern Emerg Med.

[84] Yang Z, Cai D, Sun Y (2021). Intramedullary lesion resection as an effective treatment of spinal cord microscopic polyangiitis: a case report. Neurol Sci.

[85] de Boysson H, Zuber M, Naggara O (2014). Primary angiitis of the central nervous system: description of the first fifty-two adults enrolled in the French cohort of patients with primary vasculitis of the central nervous system. Arthritis Rheumatol.

[86] Salvarani C, Brown RD, Christianson T (2015). An update of the mayo clinic cohort of patients with adult primary central nervous system vasculitis. Medicine.

[87] Salvarani C, Brown RD, Huston J (2014). Treatment of primary CNS vasculitis with rituximab: case report. Neurology.

[88] de Boysson H, Arquizan C, Guillevin L, Pagnoux C (2013). Rituximab for primary angiitis of the central nervous system: report of 2 patients from the French COVAC cohort and review of the literature. J Rheumatol.

[89] Salvarani C, Brown RD, Calamia KT (2008). Efficacy of tumor necrosis factor α blockade in primary central nervous system vasculitis resistant to immunosuppressive treatment. Arthritis Rheum.

